# Intrathecal Administration of Ziconotide as a Potential Treatment for Chronic Migraines

**DOI:** 10.7759/cureus.23714

**Published:** 2022-03-31

**Authors:** Ryan Holden, Gaurav Chauhan, Trent Emerick

**Affiliations:** 1 Anesthesiology and Perioperative Medicine, University of Pittsburgh Medical Center, Pittsburgh, USA; 2 Anesthesiology and Perioperative Medicine, University of Pittsburgh Medical Center Presbyterian, Pittsburgh, USA; 3 Pain Medicine, University of Pittsburgh Medical Center, Pittsburgh, USA

**Keywords:** intrathecal pump therapy, neuropathic pain treatment, chronic pain management, ziconotide, migraines

## Abstract

Migraine is one of the most prevalent and debilitating illnesses globally. There are multitudes of treatment options available for migraines. One of the emerging treatment options for migraine, refractory to conventional treatment modalities, is the intrathecal Ziconotide. Ziconotide (Prialt, Jazz Pharmaceuticals, Dublin, Ireland) enforces selective block of N-type calcium channels, which control neurotransmission at many synapses. Ziconotide is proposed to have efficacy for chronic neuropathic pain, with a favorable lack of tolerance and chemical dependency. Few studies in the literature report the successful resolution of migraine headaches with Ziconotide. The authors report the successful use of intrathecal Ziconotide therapy for chronic refractory migraines.

## Introduction

Migraine is one of the most prevalent and debilitating illnesses globally, affecting 18% of females and 6% of males annually. More than half of migraine patients miss work due to debilitating symptoms [1,2]. The two main types of migraines are chronic and episodic. Chronic migraine is clinically defined as 15 or more headaches per month over three months, with at least eight headaches attributable to migraine. Migraines are episodic when 0 to 14 headache days per month [1,2].

Treatment options for chronic migraines are broken down into preventive and abortive [3]. Prophylactic treatment is more effective in treating episodic than chronic migraines and includes antihypertensives, antidepressants, antiepileptics, supplements, and BOTOX injections. Abortive treatments include triptans, ergotamine derivatives, and nonsteroidal anti-inflammatory drugs [3,4].

Triptans are the gold standard of acute migraine treatment but have significant contraindications that limit their use, such as in patients with cardiovascular diseases. Another newer line of medications, Calcitonin gene-related peptide (CGRP) antagonists, do not cause vasoconstriction, making it safe for patients with migraines who cannot use triptans. CGRP receptor antagonists (gepants) have demonstrated similar efficacy and fewer side effects than triptans [5].

Ziconotide is a synthetic version of ω-conotoxin MVIIA (ω-MVIIA), a peptide found in the venom of the fish-eating marine snail. Ziconotide is composed of highly hydrophilic 25 amino acids polypeptide and has a median terminal half-life of 4.5 hours. Ziconotide has limited ability to cross the blood-brain barrier and is rapidly degraded by peptidases in the systemic circulation [6]. To optimize adequate drug delivery and reduce systemic side effects, Zicontide is administered intrathecally. This spinal route of administration permits Ziconotide to reach its maximum local concentration in a short time, which encourages a rapid onset of analgesia [6-8]. Ziconotide selectively blocks N-type voltage-gated calcium channels (VGCC), and its analgesic effect is hypothesized to be mediated by blockade of presynaptic VGCC on primary nociceptive afferents in Rexed laminae I and II of the dorsal horn. Ziconotide is commonly used in patients intolerant or refractory to treatment modalities like systemic analgesics, adjunctive therapies, or intrathecal morphine [6-8].

The most common adverse reactions of Ziconotide are dizziness (42%), nausea (30%), nystagmus (23%), confused state (25%), gait abnormal (16%), memory impairment (13%), blurred vision (14%), headache (12%), asthenia (13%), vomiting (11%) and somnolence (10%). A history of psychosis is advised to be a contraindication, although low-dose strategies have substantially mitigated but not eliminated the risk of serious neuropsychiatric side effects [8].

The authors report the successful use of intrathecal Ziconotide therapy for chronic refractory migraines. This case adds to the evidence that Ziconotide may be a useful therapeutic option for managing severe refractory migraines.

## Case presentation

The patient gave informed consent for the publication of this report. The patient, a 46-year-old Caucasian female, presented with migraines and thoracic back pain to the chronic pain clinic. The patient reported migraines that began as a teenager but intensified in 2011 and were reportedly related to family/life stressors, certain perfumes, flowers, and menstruation. Her thoracic pain started insidiously in 2016 and was unrelated to any triggering event or history of skin lesions, infections, or injury. The patient also had a past medical history of peptic ulcer disease, diverticulosis, and uterine fibroids.

The patient reported 15 to 17 headache days a month; five to eight days were migrainous headaches. These episodes were predominantly left-sided in nature and consistently occurred for greater than three months. Migraines were associated with an aura described as a “flash of light” followed by light and sound sensitivity and nausea. The patient was initially started on topiramate (100 mg daily) and naratriptan (2.5mg PRN), which failed to control her symptoms.

The patient described her thoracic spine pain as a non-radiating, aching, shooting, and burning sensation and rated it as 6-8/10 on the Numeric Rating Scale (NRS) for pain. The thoracic pain was exacerbated by lifting objects, stress, and ambulation and was alleviated by bed rest and heated water bottles. The patient reported no therapeutic benefit from over-the-counter analgesics such as acetaminophen and ibuprofen, selective norepinephrine reuptake inhibitors (duloxetine 60mg daily), opioids (hydrocodone 10mg q6hrs PRN), and physical therapy. The patient attended physical therapy twice a week for six weeks but discontinued it due to increasing thoracic pain from treatment. 

A physical exam demonstrated no sensory or motor anomalies. The patient had intact sensation, normal strength, and symmetrical reflexes in the upper and lower extremities. Magnetic Resonance Imaging (MRI) of the thoracic spine revealed a small syrinx in the mid to lower thoracic spine (T4-T8) with no other structural abnormalities. Cervical MRI reported moderate right and mild left neural foraminal narrowing, at C6-C7, due to uncovertebral arthropathy. Lumbar MRI showed mild degenerative changes with no central canal or neuroforaminal stenosis.

The patient was placed on a multimodal regimen including gabapentin (400 mg TID), tramadol (200 mg daily), and a transcutaneous electrical nerve stimulation unit for upper thoracic back pain. Over the next few months, despite being compliant with the medication, the patient complained of suboptimal pain control and reported feeling foggy and sedated in the morning, attributed to gabapentin. Gabapentin was tapered off gradually and switched to an equianalgesic dose of pregabalin (50 mg twice daily), leading to the resolution of symptoms. Nortriptyline (10 mg at bedtime) and 4% lidocaine transdermal patches were also added to the analgesic regimen for upper back pain. The patient also underwent an intercostal nerve block at the level of the T12 rib and reported minimal relief.

Over the next year, the patient sought treatment from different physicians and underwent multiple interventional procedures, including diagnostic thoracic facet blocks, thoracic medial branch radiofrequency ablation, erector spinae block, and thoracic epidurals, and reported no long-term therapeutic benefit. The patient tried medical cannabis, which helped control symptoms but was not a long-term solution because of the high cost.

Two years after the initial diagnosis and in late 2019, the patient finally consulted the authors' pain clinic. The patient reported an increase in the frequency of migraine headaches, along with thoracic pain, which significantly affected her quality of life. The patient was increasingly anxious and depressed after multiple therapeutic failures for migraines and thoracic pain. The initial focus was to provide treatment for thoracic pain, and after reviewing the history, intrathecal Ziconotide therapy was recommended to treat her refractory thoracic pain. 

**Figure 1:**
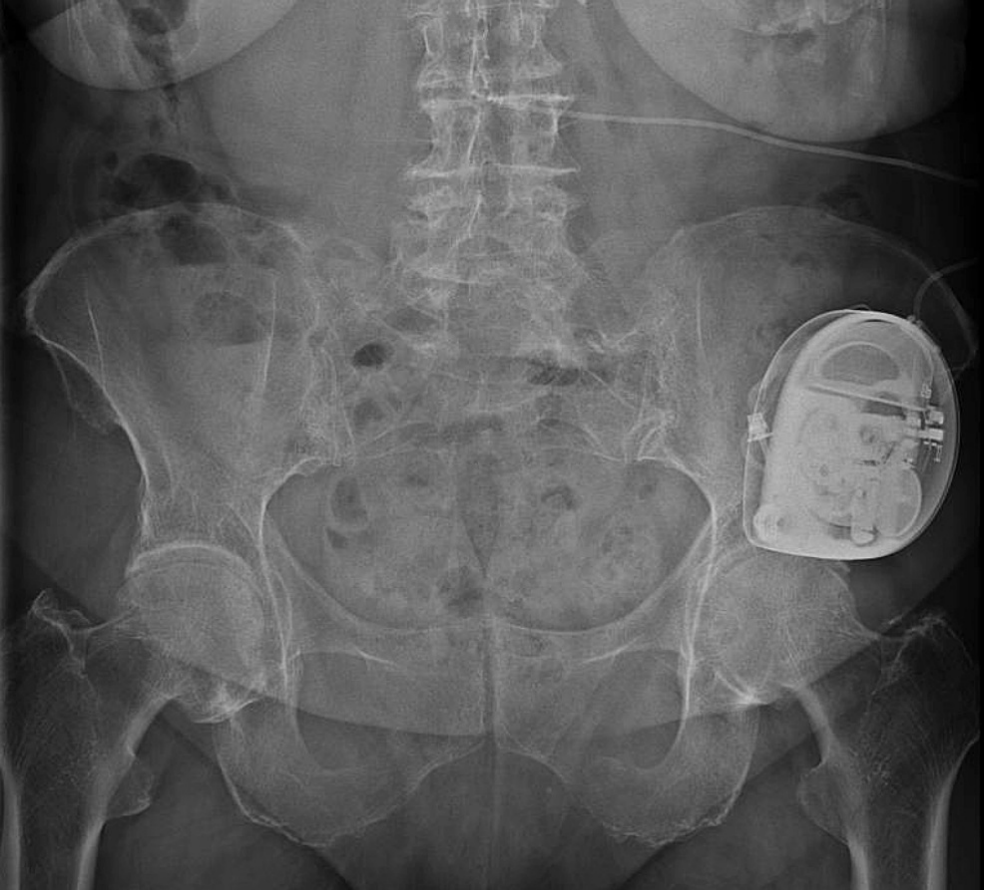
X-ray image of the pelvis depicting intrathecal pump delivering Ziconotide.

**Figure 2:**
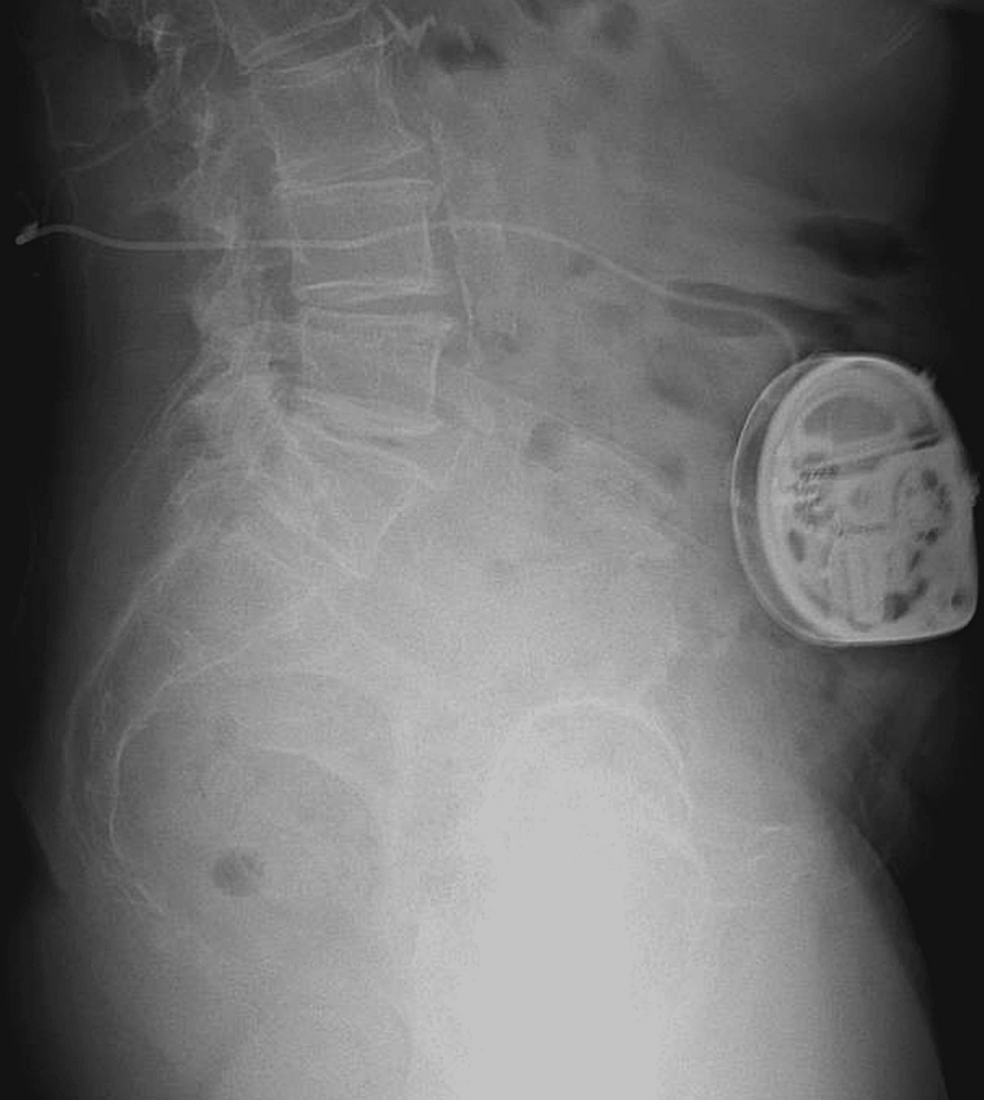
Lateral image of the intrathecal pump with pump catheter placed in intrathecal space.

The patient underwent a trial in which two micrograms of Ziconotide were administered intrathecally via a 25-gauge 3.5-inch spinal needle at L4-5 in the prone position. After the injection, the patient reported 75% relief in her thoracic pain for 30 hours. The patient also reported an immediate resolution of her migraine, which was unexpected, and lasted for 24 hours. Subsequently, the patient underwent SynchroMed II (Medtronic) intrathecal pump placed in the left abdomen without complication (Figures 1, 2).

The intrathecal catheter tip was placed in the lumbar cistern below L1. The intrathecal pump was set to deliver 2.0 mcg of Ziconotide per day.

After the intrathecal Ziconotide pump placement, the patient reported a complete resolution of her migraine but still said sub-optimal control of thoracic spine pain. In the subsequent visits, an intrathecal dose of Ziconotide was increased from 2 to 2.5 mcg/day to enhance pain control due to thoracic pain. Over the last few months, an amount of Ziconotide was finally titrated to 3.7 mcg/day to control her thoracic pain optimally. At 12 months, post-IT pump implant, the patient now reports continued resolution of her atypical facial pain and a significant decrease in her thoracic pain (2-4 on an 11-point NRS scale).

## Discussion

Intrathecal administration of Ziconotide is reported to be efficacious in treating many different types of chronic pain diagnoses such as chronic pain in cancer patients and refractory pain in acquired immunodeficiency syndrome (AIDS) patients, trigeminal neuralgia, and persistent idiopathic facial pain (PIFP) [9]. Unlike intrathecal opioids, Ziconotide is not associated with catheter tip granuloma, can be discontinued abruptly without concerns for withdrawal, and is not related to the risk of respiratory depression [9,10]. Intrathecal Ziconotide is not associated with the development of tolerance. The side effect profile of Ziconotide includes psychiatric symptoms such as confusion, memory impairment, speech disorder, hallucinations, paranoid reactions, hostility, delirium, psychosis, suicidality, and manic reactions [8,9].

Ziconotide has a volume of distribution that equals the total estimated cerebrospinal fluid (CSF) volume. Even a low intrathecal dose can spread from the lumbar region to the brain and brain-stem tissue [8,9]. Ziconotide blocks presynaptic N-type voltage-sensitive calcium channels in laminae I and II of the dorsal horn of the spinal cord. It is hypothesized that Ziconotide achieves its analgesic effect by blocking signal propagation from primary afferents to ascending pathways. However, the precise mechanism of action of intrathecal Ziconotide is still unknown [7-9].

There are case reports in the literature that document the successful use of intrathecal Ziconotide for chronic trigeminal neuralgia and facial pain [10-12]. A literature search produced two isolated case reports of trigeminal neuralgia improving with intrathecal Ziconotide. In the first case report, intrathecal Ziconotide therapy was successfully used for pain in the distribution of the maxillary branch of the trigeminal nerve. The patient reported a decreased pain score from 9/10 to 3-4/10 on an NRS for pain, consistent at her five-month follow-up [11]. In the second case, a 59-year-old female had a single-shot intrathecal ziconotide trial of 1 μg at T12-L1, which decreased her pain due to trigeminal neuralgia from 9/10 to 6/10 [12]. One case report of a patient who experienced temporary albeit complete resolution of her facial pain (PIFP) upon a single injection of the intrathecal trial of Ziconotide a the L3-L4 level [13]. In one case of a 59-year-old female, Ziconotide therapy at the rate of 1 microgram per day was successful in effectuating complete resolution of chronic refractory migraine headaches [10].

In our case report, the overall change in ziconotide dosing over the preceding 12 months is consistent with the considerable dosing variation seen with Ziconotide between patients. The current dose is considerably less than the maximum recommended amount of 19.2 mcg/day. The dosing increases occurred much less frequently than the expert consensus guidelines of no more than once per week, at 0.5 mcg/day or less [14,15]. The dose increases seen over the preceding 12 months with this patient are consistent with consensus guidelines for ziconotide titration and likely represent the patient's desire for optimized pain control due to a second pain generator to match the level of relief she achieved single bolus trial [14,15]. In the case report published by Narain et al., the intrathecal catheter was placed at the level of the ninth thoracic spine [10]. The intrathecal catheter was placed below the conus medullaris in the lumbar intrathecal space in this case report. This difference in placement is vital because it shows that pain control in migraines can be affected even from a catheter tip in the lumbar region compared to the thoracic region. The insertion of a catheter tip below the conus medullaris improves safety compared to a catheter tip located above the same. In addition, the doses for the intrathecal pumps differ between studies. Furthermore, Narain et al. employed bolus/flex dosing due to the patient's symptoms of spasticity induced by multiple sclerosis [10]. The patient, in this case report, had a dosing strategy scheduled for flex doses every four hours such that the 1 µg/day dose of Ziconotide was achieved. This study started the patient at two µg/day with a continuous dosing regimen. Both patients exhibited total migraine relief despite the differences in dosing technique, which suggests that symptom relief may be due to the total dosage of Ziconotide rather than the number of dosing intervals.

In conjunction with other case studies, this report provides evidence that intrathecal administration of Ziconotide may be a potential therapeutic option for managing severe refractory migraine facial and cranial pain. This report also documents that the catheter tip in a lower, more accessible location and relatively safer lumbar space instead of the thoracic region is a viable option.

## Conclusions

Intrathecal administration of ziconotide is a potential long-term therapeutic option for the treatment of chronic migraines as well as facial/cranial associated pain. Symptom relief due to ziconotide may be due to total dosage rather than dosing intervals. The catheter tip of the intrathecal ziconotide pump in a lower, more accessible location and relatively safer lumbar space instead of the thoracic region is a viable analgesic option for atypical facial pain.
